# Indigenous data governance approaches applied in research using routinely collected health data: a scoping review

**DOI:** 10.1038/s41746-024-01070-3

**Published:** 2024-03-15

**Authors:** Teyl Engstrom, Elton H. Lobo, Kristie Watego, Carmel Nelson, Jinxiang Wang, Howard Wong, Sungkyung Linda Kim, Soo In Oh, Michael Lawley, Alain-Dominique Gorse, James Ward, Clair Sullivan

**Affiliations:** 1 Queensland Digital Health Centre, Centre for Health Services Research, The University of Queensland, Herston, QLD Australia; 2 Institute for Urban Indigenous Health, Windsor, QLD Australia; 3 Poche Centre for Indigenous Health, The University of Queensland, Herston, QLD Australia; 4 CSIRO, Herston, QLD Australia; 5 Queensland Cyber Infrastructure Foundation, St Lucia, QLD Australia; 6 Royal Brisbane and Women’s Hospital, Herston, QLD Australia

**Keywords:** Medical ethics, Medical research

## Abstract

Globally, there is a growing acknowledgment of Indigenous Peoples’ rights to control data related to their communities. This is seen in the development of Indigenous Data Governance standards. As health data collection increases, it’s crucial to apply these standards in research involving Indigenous communities. Our study, therefore, aims to systematically review research using routinely collected health data of Indigenous Peoples, understanding the Indigenous Data Governance approaches and the associated advantages and challenges. We searched electronic databases for studies from 2013 to 2022, resulting in 85 selected articles. Of these, 65 (77%) involved Indigenous Peoples in the research, and 60 (71%) were authored by Indigenous individuals or organisations. While most studies (93%) provided ethical approval details, only 18 (21%) described Indigenous guiding principles, 35 (41%) reported on data sovereignty, and 28 (33%) addressed consent. This highlights the increasing focus on Indigenous Data Governance in utilising health data. Leveraging existing data sources in line with Indigenous data governance principles is vital for better understanding Indigenous health outcomes.

## Introduction

The adoption of electronic medical records (EMRs) across healthcare systems is rapidly accelerating, particularly in Asia and Europe^1^. EMRs play a crucial role in capturing and storing a wide range of health data, encompassing medical history, clinical information, and personal details^2,3^. This wealth of health data has the potential to improve patient care and generate value within healthcare organisations^4^. The value of EMRs within healthcare organisations is created by enabling clinicians to access patient records from within the same system at any time, streamlining care and facilitating innovation^5^. Further work is needed to ensure that EMR systems prioritise patient-centeredness, delivering equitable benefits at a population health level while enabling seamless data sharing across multiple agencies. Moreover, enhancing the utilisation of this data in technologies like Artificial Intelligence and Machine Learning is essential. Despite the considerable advantages presented by the electronic collection and sharing of patient information between service providers and clinicians, there are still challenges, particularly regarding privacy, security and governance^6^. These challenges are further exacerbated for many Indigenous people^7^, for whom the willingness to embrace new technology may be tainted by past experiences of unethical data collection and management, including through research, stemming from inherent racism biases and failure to recognise and respect the rights of Indigenous peoples^8^.

The Declaration on the Rights of Indigenous Peoples (UNDRIP) was adopted by the United Nations in 2007 to establish universal minimum standards for the rights of Indigenous Peoples^9^. The UNDRIP Article 31 specifically includes standards for Indigenous Peoples to exercise control over intellectual property pertaining to their communities, lands, and resources^10^. In addition, Article 18 addresses the data rights of Indigenous Peoples, emphasising their inclusion in decision-making processes that impact their rights in alignment with their own established procedures^8^. These standards offer comprehensive approaches to managing Indigenous Peoples’ data beyond mainstream notions of research processes, knowledge generation and intellectual property^11^.

Indigenous Data Governance (IDG) and Indigenous Data Sovereignty (IDS) are relatively new methodologies increasingly advocated for in Indigenous communities to be able to govern the collection, analysis and interpretation of data that relates to their sovereign rights. These principles have been developed largely from standards contained within the UNDRIP, and generally, they reaffirm the rights of Indigenous Peoples to control the collection, access, analysis, interpretation, management, dissemination, and reuse of data relating to their communities^12^. The implementation of Indigenous data sovereignty revolves around two fundamental principles: (i) the sovereignty of Indigenous People concerning data pertaining to them, regardless of its location or custodian, and (ii) the entitlement to access the data necessary for Indigenous Peoples’ nation-building efforts^13^. While standards exist to advocate for Indigenous data sovereignty, the practical application of these standards in research activities involving data from Indigenous communities remains unclear.

This prompts the research question: What are the current practices used in research for governing Indigenous Peoples’ routinely collected health data? The primary objective of this study is to systematically review the data governance approaches employed when using routinely collected health data for Indigenous Peoples for research purposes. The secondary objective was to understand the advantages and challenges of using this data for research, which is particularly relevant for Indigenous Peoples given the burden of research on Indigenous Peoples, who are some of the most researched groups in the world^14^.

## Methods

This scoping review was guided by Arksey and O’Malley’s framework for scoping studies^15^. In addition, the study selection and presentation followed the PRISMA extension for scoping reviews (PRISMA-ScR) guideline^16^. The PRISMA-ScR checklist is available in Supplementary Table 1. The scoping review methodology was selected for this study because, unlike systematic reviews, it is particularly effective in synthesising research and mapping literature in areas that were either not extensively reviewed or are complex and diverse in nature^17^.

### Search strategy

We searched five electronic databases (PubMed, EMBASE, CINAHL, Web of Science, ATSIHealth), including one database which focuses on Aboriginal and Torres Strait Islander health studies (ATSIHealth), for materials published from 2013 to 6 December 2022. A professional librarian provided help to develop the search strategy; full search terms are available in Supplementary Table 2.

The search strategy was designed to identify papers that included: (1) Indigenous Peoples across various countries worldwide and (2) Access to routinely collected health data. To identify studies which included Indigenous Peoples, we used subject headings such as ‘Health Services, Indigenous’, ‘Indigenous People’s’, ‘United States Indian Health Services’ and related free text searches. Similarly, studies which accessed routinely collected health data were found using subject headings such as ‘Medical Record Linkage’, ‘Routinely Collected Health Data’ and related free text searches.

### Study selection

Title and abstract review inclusion and exclusion criteria were drafted, and a sample of 50 papers were reviewed by two researchers (T.E. and J.W.) to refine and agree on the final criteria. The same sample of 50 papers was reviewed by the other researchers (H.W. and S.K.), and conflicts were discussed to ensure all reviewers had a consistent understanding of the criteria. Inclusion and exclusion criteria are described in Table 1.

**Table 1:** Title and abstract review inclusion and exclusion criteria

Criteria	Include	Exclude
Study type	Peer-reviewed original research paper	Protocol, review, commentary, etc.
Study population	Access data for Indigenous Peoples predominantly (90%+)	Accessed data for the general population or some other group not defined by Indigenous status (even if it includes some Indigenous People)
Data source	Accessed data from ieMR or administrative dataset that was already collected as part of routine healthcare	Used/collected custom data specifically for the purpose of this study only
Study outcome	Studies of health outcomes, access, etc., or implementation studies related to these outcomes	Studies looking at data quality only, data governance only or data principles only
Level of data	Personal individual-level health data	Population-level/summary health data only

The title and abstract review of each article was performed by two independent researchers, randomly assigned by Covidence to members of the research team (T.E., J.W., H.W., S.K.). Conflicts were resolved through group discussions with at least two researchers.

Full-text review was also conducted by two researchers independently, randomly allocated by Covidence to research team members (T.E., J.W., H.W., S.H., S.K., S.O.), with conflicts resolved through a group discussion with at least two researchers. Papers were excluded if: (1) could not locate a full-text article; (2) full text not available in English; or (3) not peer-reviewed original research article; or (4) not focused on Indigenous People (at least 90% of study participants); or (5) did not use routinely collected health data; or (6) study outcome was not a health outcome; or (7) did not use personal level health data.

Cohen’s Kappa was extracted from Covidence, and a weighted average was calculated to compare inter-rater reliability for both stages of the review.

### Data extraction

Study characteristics, Indigenous data governance approaches and advantages and disadvantages of using routinely collected health data were extracted from the included papers. One reviewer (T.E.) developed a data extraction template in Covidence and tested it with four other reviewers (H.W., J.W., S.K., S.O.) independently extracting five articles each. Conflicts were discussed, and refinements were made to the data extraction template. Double data extraction was then completed by six reviewers (T.E., H.W., J.W., S.K., S.O., E.L.). One reviewer (T.E.) resolved conflicts for consistency.

A risk of bias assessment was not conducted as part of this scoping review, as the purpose is to examine Indigenous data governance practices reported, not to report on the outcomes of the studies.

### Data analysis

Data extracted from Covidence was exported into a spreadsheet. The study characteristics were analysed using descriptive statistical techniques. A table was produced summarising the number of studies in each category. For the extraction of qualitative data, a thematic analysis approach was employed, following the methodology of Braun and Clarke^18^. This thematic analysis methodology involved two reviewers (E.L., H.W.) familiarising themselves with the data to generate coding elements and then iteratively comparing these coding elements to identify recurrent themes and subthemes.

The frequency of the main Indigenous data governance approach being reported in the included studies was summarised in a table. The Indigenous data governance approaches, advantages and disadvantages described in the studies were distilled into a checklist of considerations for using Indigenous Peoples’ routinely collected health data for research. The table was structured according to the horizons of digital transformation in health^19^, a commonly used framework in digital health. The horizon names were amended to focus on data selection, access and use.

## Results

**Fig. 1:**
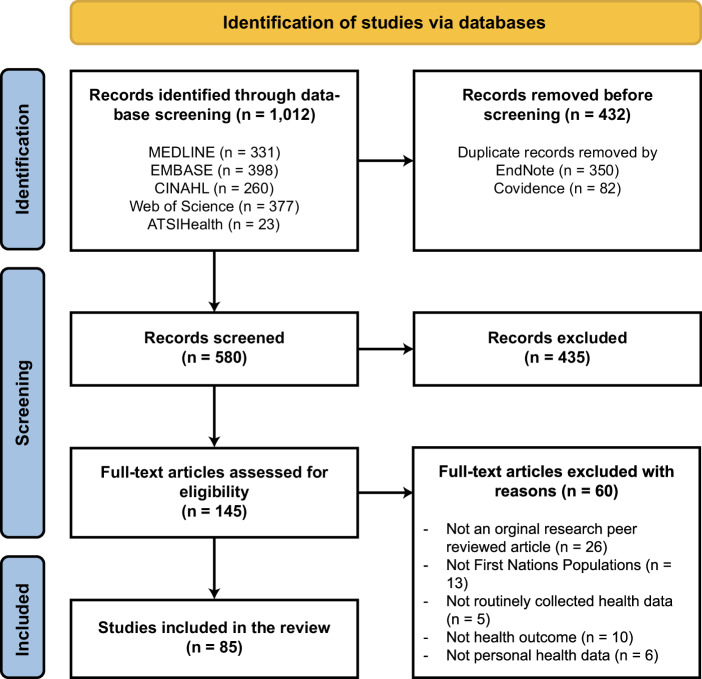
PRISMA study selection diagram. *From:* Page MJ, McKenzie JE, Bossuyt PM, Boutron I, Hoffmann TC, Mulrow CD, et al. The PRISMA 2020 statement: an updated guideline for reporting systematic reviews. BMJ 2021;372:n71. 10.1136/bmj.n71. For more information, visit http://www.prisma-statement.org/.

The combined searches identified a total of 1012 articles; after removing duplicates using EndNote and Covidence, 580 unique articles remained. After the title and abstract screening, 145 articles were included for full-text retrieval. Reviewer agreement was moderate for title and abstract screening (*κ* = 0.58). All full-text articles were found and assessed for eligibility, which resulted in 85 articles being included (Fig. 1). The reviewers had a substantial agreement on study inclusion (*κ* = 0.63).

### Characteristics of included studies

The characteristics of the studies included in this review are summarised in Table 2, and the details of each study are available in Supplementary Table 3. The included studies were published between 2013 and 2022. Studies were carried out in four countries, including Australia (*n* = 38; 44.7%), the United States (*n* = 25; 29.4%), Canada (*n* = 19; 22.4%), and New Zealand (*n* = 3; 3.5%).

**Table 2:** Study characteristics of included studies

Characteristics	Number of studies	
	*n*	%
*Published Year*		
2021–2022	25	29.4
2019–2020	27	31.8
2017–2018	11	12.9
2015–2016	8	9.4
2013–2014	14	16.5
*Country*		
Australia	38	44.7
United States	25	29.4
Canada	19	22.4
New Zealand	3	3.5
*Region study was conducted*		
Rural	45	52.9
Urban	12	14.1
Both	24	28.2
Not described	4	4.7
Indigenous group/s*		
Aboriginal Australian	38	44.7
Torres Strait Islander	24	28.2
Alaska Native	19	22.4
American Indian	18	21.2
First Nations living in Canada	14	16.5
Inuit	4	4.7
Māori	2	2.4
Métis	2	2.4
Pacific Islander	2	2.4
*Gender*		
All	75	88.2
Female	10	11.8
*Outcome measured*		
Healthcare Utilisation and Access	24	28.2
Maternal and Child Health	17	20.0
Chronic Diseases and Comorbidities	15	17.6
Infectious Diseases	10	11.8
Mental Health and Suicide Risk	8	9.4
Public Health and Prevention	6	7.1
Dental Health	2	2.4
Other Health Conditions	7	8.2

Amongst the 85 articles included in this review, 82 articles reported on the number of participants, ranging from 8 to 138,551. One article considered the number of visits (i.e., 5373) of the target population, while another included 29 Aboriginal Community Controlled Health Services representing 34 individual clinics and 5 clinical hubs. One article did not describe the number of participants/visits. A majority of articles considered all genders (*n* = 70; 82.4%), while 10 studies focused on women only (11.8%). The articles included participants of one or more Indigenous backgrounds, with a majority being Aboriginal Australian (*n* = 38; 44.7%), Torres Strait Islander (*n* = 24; 28.2%), Alaska Native (*n* = 19; 22.4%), American Indian (*n* = 18; 21.2%) and First Nations living in Canada (*n* = 14; 16.5%). More than half (*n* = 45; 52.9%) of the studies focused on populations in rural or regional populations, 14.1% considered urban areas only, and 28.2% considered both.

The studies examined one or more health outcomes including healthcare utilisation and access (*n* = 24; 28.2%), maternal and child health (*n* = 17; 20%), chronic diseases and comorbidities (*n* = 15; 17.6%), infectious diseases (*n* = 10; 11.8%), mental health and suicide risk (*n* = 8; 9.4%), public health and prevention (*n* = 6; 7.1%), dental health (*n* = 2; 2.4%), and other health conditions (*n* = 7; 8.2%).

### Indigenous data governance

The frequency of Indigenous data governance approaches described in the studies is included in Table 3. A checklist of considerations for using Indigenous Peoples routinely collected health data for research synthesised from the included studies is shown in Table 4, structured by the horizons of digital transformation^19^.

**Table 3:** Frequency of Indigenous Data Governance Approaches Described in Included Studies

Indigenous data governance approach	Described in study *n* (%)	
	Yes	No
Data sovereignty	35 (41.2%)	50 (58.8%)
Approach to consent	28 (32.9%)	57 (67.1%)
Indigenous Peoples and Communities involved in research	65 (76.5%)	20 (23.5%)
Ethics approval	79 (92.9%)	6 (7.1%)
Indigenous guiding principles	18 (21.2%)	67 (78.8%)
Advantages of using routinely collected health data	28 (32.9%)	57 (67.1%)
Disadvantages of using routinely collected health data	39 (45.9%)	46 (54.1%)

**Table 4:** Considerations for selecting, accessing, and using Indigenous Peoples Routinely Collected Health Data for Research

Horizon	Action
Horizon 1: Data selection	• Indigenous People should be reliably identified in the data • Data should be of sufficient quality and completeness • Using routinely collected health data should alleviate potential participant burden and cost of collecting new data • Using routinely collected data should allow in-depth analysis not otherwise possible—e.g. longitudinal analysis, or linking across multiple sources to provide a more wholistic view
Horizon 2: Data access and sovereignty	• Seek appropriate Indigenous Community and Organisational approvals to access the data • Employ an appropriate model of consent—Individual, Community, or waiver of consent • Get formal ethics approval from an Indigenous-specific ethics committee, or an ethics committee that has experience with Indigenous research • Ensure Indigenous Peoples and Communities maintain sovereignty over their data throughout research process • Consider additional requirements for health departments or hospitals to maintain control of data
Horizon 3: Research and translation	• Identify appropriate Indigenous guiding principles to inform the research • Research team leadership and study authors should include Indigenous Peoples and Indigenous organisations • Receive approval from Indigenous Leaders, Communities, and Organisations to conduct research • Research questions and study design should be Indigenous led • Incorporate Indigenous perspectives into interpreting research findings

#### Indigenous data sovereignty

Data sovereignty aspects were described in 34 (40%) of the studies. Eighteen studies outlined the requirement of the state health services to maintain control of the data^20–36^, while 15 studies outlined Indigenous Peoples or Communities sovereignty over their own data^27,31,32,37–48^. Fourteen (16.5%) studies described the researchers’ inability to share data publicly^24,26,33–35,41,45–52^ due to privacy and ethical restrictions^31,35,41,47,49,51,52^. However, in 11 (12.9%) studies, the researchers described the data can be obtained upon reasonable request^30,31,35,37,41,47–52^, subject to additional institutional^23,26–36,44,45,49,52,53^ or Tribal^27,31,32,37,41–46,48^ approvals, and/or compliance with privacy policies^26,27,34,46,47^. Furthermore, researchers had considered the use of de-identified data to analyse and present information with the intention to promote the anonymity of the Indigenous People whose data was accessed in the research study^20–27,29–33,36,38,41,46,48,50,54–72^.

In addition to the approvals, studies highlight that data collected should be securely stored in various repositories^21,31,32,50^ and regulated by organisations such as Tribal health Organisations^40^, healthcare/government departments^38,39^, and data custodians^25,30^. Access to the data is restricted to research investigators^21^ or people who meet prespecified criteria for data access^27,31,35,47,50,51^. This information was most commonly included in a Data Availability Statement, which is increasingly being required by journals, and hence was more prevalent in recent studies (10% of studies published 2013–2015 vs. 63% of studies 2020–2022).

In terms of data sovereignty principles and access, the procedures vary with individual context. For example, in Manitoba, approval from specific entities is required to access data^27,31–34^. The Navajo Nation^41^ and Western Australia^24,35,47^ also have specific processes for data access, while at the Sioux Lookout, access and management of data need to be conducted in accordance with the principles of ownership, control, access and possession (OCAP)^40^.

#### Approach to consent

The majority of studies (*n* = 57, 67.1%) did not describe how consent was approached. Of the 28 (32.9%) studies which did include this information, nine studies obtained consent from individual participants^28,31,61,65,73–77^. Fifteen studies employed a waiver of consent to access participants' health data^21,23,24,33,36,37,41–43,47,49,55,63,66,71^. Another approach to consent was to obtain permission from Community Leaders^46^ or Community Organisations (Aboriginal Community Controlled Health Services) involved^48^. Furthermore, in studies that required follow-up care^78^ or further review of specific individuals’ documents^79^, additional consent was sought from the participants at that stage.

#### Involvement of indigenous community and people in research

The researchers in 65 (76.5%) studies described various measures undertaken to ensure their research was conducted with the involvement and approval of the Communities they worked with. In several studies, researchers obtained approvals from Indigenous Leaders^32,33,55,74,80^, institutional organisations^34,37,39,44,52,56,59,61–63,74,81–83^, and Tribal health Organisations^21,23,31–33,35,37,39,42–45,47,50–53,56,59,61–63,68,72,81–89^ to commence, undertake and/or disseminate findings at various stages of the research study. In addition, partnerships were established with various Indigenous Leaders^34,55,61,74^, Communities^22,30,31,34,44,49,52,62,66,69,73,75,79,80,90^ and Organisations^21,23,26,29,31,33,35,39–41,46–48,50,53,55,56,59,66,67,69,71,73–75,77,79,80,85,87,88,91–93^ to incorporate their perspectives and ensure cultural relevance^23,34,50,51,56,62,73,79^.

In one study by Struck, et al.^56^, the researchers described that the research needs to be conducted in the spirit of truth and reconciliation with recognition of the harms conducted to Indigenous People. By focusing on transparency, mutual respect, and maintaining a shared understanding of Indigenous data,^56,81^ it may be possible to achieve deep trust^81^ and respectful collaboration with Indigenous People^61^. While in some studies^21,23,24,33,36,37,41–43,47,49,55,63,66,71,81^ researchers received approval for a waiver of informed consent, efforts were made to maintain transparency and trust between researchers and Indigenous Communities^81^.

#### Indigenous organisation author affiliations

The inclusion of Indigenous Organisations in the research study was evident in 60 (70.6%) studies where one or more co-authors were affiliated with Indigenous Health, Research or Community Organisations. These co-authors participated in the design, development, data collection and analysis of the research study^24,35,42,60,65,71^.

#### Indigenous ethics approval

Thirty-five (41.2%) studies reported receiving ethics approval from an Indigenous-specific ethics committee for their study^23,24,29,35–37,39–48,56,59,61,63,64,68,69,80,82–87,90,92–95^. Forty-three (50.6%) studies detailed receiving ethical approval to conduct their study from a non-Indigenous ethics committee. Six studies did not describe whether ethical approval was received, and one study stated ethical approval was not required.

#### Indigenous guiding principles

Eighteen (20%) studies described using Indigenous guiding principles to inform their research^22,31,32,34,35,40,46,48,50,52,53,56,72,73,78,89,92^. For example, in Canada, several studies focused on the use of OCAP (ownership, control, access, and possession)^31,32,40,46,50,92^ and OCAS (ownership, control, access, and stewardship)^56^ principles in Indigenous health research. These principles were followed throughout the study^31,32^ to ensure governance of Indigenous data^46^. One study also described the inclusion of Chiefs of Ontario First Nations Data Governance Committee and the Grand Council Treaty towards the review of the study’s compliance with the OCAP principle^50^, while another study was supervised, and the data were maintained by the Sioux Lookout First Nations Health Authority in accordance with the OCAP principle^40^. Other studies focused on including several ethical and scientific standards from the various Canadian Institutes (Canadian Institutes of Health Research, Natural Sciences and Engineering Research Council of Canada, and Social Sciences and Humanities Research Council of Canada)^73^. In particular, Section 6 of the Tri-Council Policy Statement regarding the Ethical Conduct for Research Involving Humans, that involves First Nation, Métis or Inuit People^26,89^. Moreover, one study by Pena-Sanchez, et al.^22^ utilised the Indigenous medicine wheel as its foundational framework, supported by cultural safety and patient-oriented research principles. It was guided by two specific Calls to Action from the Truth and Reconciliation Commission (TRC) of Canada. TRC 18 emphasised acknowledging the then-current state of Aboriginal health and implementing the healthcare rights of Aboriginal Peoples, and TRC 19 called for establishing goals in consultation with Aboriginal Communities to identify and address health outcome disparities. Additionally, one study co-developed its protocol with the Isumataiit Sivuliuqtii, ensuring a foundation grounded in Inuit ways of knowing (Inuit Qaujimajatuqangit)^34^.

In the United States, one study focused on promoting trust and respectful collaboration with the Indigenous People concerning research participation and patient confidentiality^61^. While, in Australia, four studies emphasised compliance with the National Health and Medical Research Council (NHMRC) guidelines for ethical conduct in Aboriginal and Torres Strait Islander health research^35,52,72,78^. Another Australian study^72^ was driven by shared values such as spirit and integrity, reciprocity, respect, equity, cultural continuity, and responsibility in all network activities.

### Advantages and challenges of using routinely collected Indigenous Peoples’ health data

#### Advantages of using routinely collected Indigenous Peoples’ health data

Out of the 85 articles, twenty-eight (32.9%) of them discussed the benefit of using routinely collected Indigenous data in research in terms of enhanced efficiency and inclusivity while minimising biases and participant burden. One of the key benefits is that by leveraging existing data sources^30,37^ and linking them together^23,29,34,36,49,70,96^, researchers are able to access a wealth of information^23,28,30,34,36,37,56,62,70,75,95–97^ without requiring additional input from participants. This minimises the participant burden^72^ and reduces reporting and recall bias^33,56^, while also making the research process more efficient^87^. Moreover, it can also provide data that is broadly representative of the Indigenous Communities^75^. As a result, the studies can achieve more robust and representative findings.

Another significant benefit lies in the ability to extract comprehensive and detailed information on patients’ diagnoses, treatments, follow-up care, and relevant outcomes^45,59,97^. Researchers can utilise this data, which is often underutilised^69^, not only to examine high-risk populations^49,59,96^ and health trends of Indigenous People^28,29,34,36,37,48,56,70,72,75,85,88,96,97^ but also to monitor service utilisation^34^, interventions^20^ and outcomes^69^. These are essential for strategic planning and operational decision-making in healthcare services^29^. In addition, EMRs can allow for improved data validity and reliability^87^ while also automating data collection and analysis tasks^48,65^, which enhances the sustainability of surveillance systems^48^. The automation of these processes provides a significant advantage to researchers over the use of manual procedures^65^. This increases the efficiency and longevity of research projects, allowing them to have a lasting impact even beyond their initial funding period^48^.

#### Challenges of using routinely collected Indigenous Peoples’ health data

Several reported challenges and potential biases in using healthcare data were identified in 39 (45.9%) studies included in this review. The most significant challenge reported in the included studies is regarding data completeness^85^. Incomplete health data, including demographics and family variables^39^, physiological and lifestyle measurements^23,32,40^, laboratory report^31,50,74,87^, disease history and severity^58,71,92^, costs^40,50,97^, socioeconomic status^23^, risks^51,77^, charts^53^, health service utilisation^24,46^, diagnosis and treatment^39,40,49,55,80^, discharge abstracts^21^, and critical social and cultural dimensions^25^, were reported as either missing, underestimated, not recorded, or inaccessible. In one study, the researchers reported missing data ranging from 0 to 15.8% depending on the database^98^, while in another study, 9% of age and sex distribution data was considered to be missing^52^. The incompleteness of data was reported to be a consequence of high population mobility^54,65^, unclear clinical catchments^54^, consults in other health services^67,80^, availability of other non-department of health services^54,99^, unclear definitions used in the storage of data^45,47,53,90,99^, the inability to contact participants^69^ and/or limited medical consultations^24,36,44,100^. For example, one study described that patients who seek care outside the Indian Health Service (IHS) would not have their data recorded in the National Patient Information Reporting System^99^. This would result in a small sample size^85^, potential coding errors^75^ and inability to provide accurate estimates regarding an outcome^24,31,36,38,50–52,54,65,77,92,99^.

Data quality was identified to be another significant challenge reported in the included studies. From the included studies, it is evident that the health data is entered by the clinicians and is reliant on the consistency and quality of clinician recording^54,65,71^; often criticised for its dependency on clinicians^41^. However, this is an ongoing challenge, especially for new staff, who need to quickly learn the system and perform the necessary actions, thus affecting the accuracy and comprehensiveness of data collection^91^. The challenge of data quality may also be because of the limited scale of data^25,39^, generalisability^87^ and misrepresentation or misclassification of data^22,47,59,79^ that could lead to bias^22,33,45,62,88^. To mitigate these issues, researchers have looked towards tracing individuals through the system and by manually verifying the data^63^; however, they have been unsuccessful due to limited access^30^.

## Discussion

This review sheds light on Indigenous data governance approaches employed by researchers when accessing Indigenous Peoples’ routinely collected health data. The findings reflect that Indigenous Data Governance (IDG) is an emerging area with inconsistent reporting of these approaches. Some elements of IDG, such as ethical approval and the involvement of Indigenous Peoples in research, were widely reported, while others, such as how data sovereignty was maintained and the use of Indigenous guiding principles, were less often reported. We propose that reporting on IDG approaches provides readers with confidence that the research was conducted ethically. A reporting guideline for research using Indigenous Peoples’ routinely collected health data may be useful to encourage the explicit and consistent inclusion of IDG approaches.

The benefits of utilising routinely collected health data for research are widely recognised to enhance healthcare efficiency and delivery^101^. However, its use in research poses significant ethical challenges related to patient privacy and data access, especially for Indigenous Peoples^102,103^. Consequently, Indigenous data governance is crucial to ensure the power, authority, access to, ownership and use of data is maintained by Indigenous Populations^104^. While the implementation of such approaches requires time, resources, education, and planning, when properly executed, it can serve as a driver for Indigenous-led strategic planning and decision-making in public health^105^. These approaches can help develop deep trust^81^ and respectful collaboration with Indigenous People^61^ through transparent, mutual respect and shared understanding of Indigenous data^56,81^. Including Indigenous Community Leaders and People can ensure cultural appropriateness in the process of strategic planning and operational decision-making within healthcare services^23,34,50,51,56,62,73,79^.

Indigenous Peoples are considered to be some of the most researched groups in the world^14^, which has put a significant burden on these Communities to share information about their health and participate in trials. Utilising routinely collected health data provides an opportunity to conduct important research without the need to burden populations through additional data collection^72^. Routinely collected health data can make the research process more efficient and cost effective^87^, it can also enable comparison or follow-up across longer periods of time and access to more people than would otherwise be practical^48^. There are well-documented limitations in the quality and completeness of routinely collected health data, the most significant being the inaccurate identification of Indigenous People^29,68^. Researchers should consider these factors when deciding whether utilising Indigenous Peoples’ routinely collected health data is appropriate for their research.

Despite using rigorous methods to understand the approaches to Indigenous data governance in healthcare, this study has its limitations. The research study incorporated the ATSIHealth database which focuses on Aboriginal Australian and Torres Strait Islander, as well as other international databases, but did not include databases specific to other Indigenous groups. While it can be argued that the research may have potential biases, the authors included research assistants from Canada and the USA and ensured a comprehensive set of search terms to encompass the diverse Indigenous Communities. This ensured thoroughness in extracting data from various health databases.

### Supplementary information

Supplementary Tables

## Data Availability

The data that support the findings of this study are available from the corresponding author upon request.

## References

[1] Dendere R (2019). Patient portals facilitating engagement with inpatient electronic medical records: a systematic review. J. Med. Internet Res..

[2] Li Y (2020). BEHRT: transformer for electronic health records. Sci. Rep..

[3] Dash S, Shakyawar SK, Sharma M, Kaushik S (2019). Big data in healthcare: management, analysis and future prospects. J. Big Data.

[4] Pastorino R (2019). Benefits and challenges of Big Data in healthcare: an overview of the European initiatives. Eur. J. Public Health.

[5] Chan SCC, Neves AL, Majeed A (2023). Electronic health records: don’t underestimate the importance of implementation and training. Br. Med. J..

[6] Barnaghi, P. et al. in *Building the Hyperconnected Society-Internet of Things Research and Innovation Value Chains, Ecosystems and Markets* 221–260 (River Publishers, 2022).

[7] United Nations. *Who are indigenous peoples?*. https://www.un.org/esa/socdev/unpfii/documents/5session_factsheet1.pdf (n.d.).

[8] Rainie, S. C. et al. in *The State of Open Data: Histories and Horizons* (eds T. Davies, S. Walker, M. Rubinstein, & F. Perini) 300–319 (Cape Town and Ottawa: African Minds and International Development Research Centre, 2019).

[9] United Nations. *United Nations Declaration On The Rights Of Indigenous Peoples.* https://social.desa.un.org/issues/indigenous-peoples/united-nations-declaration-on-the-rights-of-indigenous-peoples (2007).

[10] Carroll SR, Herczog E, Hudson M, Russell K, Stall S (2021). Operationalizing the CARE and FAIR Principles for Indigenous data futures. Sci. Data.

[11] Carroll SR (2020). The CARE principles for indigenous data governance. Data Sci. J..

[12] Walter M (2021). Indigenous data sovereignty in the era of Big Data and Open Data. Aust. J. Soc. Issues.

[13] Bodkin-Andrews, G., Walter, M., Lee, V., Kukutai, T. & Lovett, R. *Delivering Indigenous Data Sovereignty*. https://aiatsis.gov.au/publication/116530 (2019).

[14] National Health and Medical Research Council. (2018). Ethical conduct in research with Aboriginal and Torres Strait Islander Peoples and communities: Guidelines for researchers and stakeholders.

[15] Arksey H, O’Malley L (2005). Scoping studies: towards a methodological framework. Int. J. Soc. Res. Methodol..

[16] Tricco AC (2018). PRISMA extension for scoping reviews (PRISMA-ScR): checklist and explanation. Ann. Intern. Med..

[17] Pham MT (2014). A scoping review of scoping reviews: advancing the approach and enhancing the consistency. Res. Synth. Methods.

[18] Braun V, Clarke V (2006). Using thematic analysis in psychology. Qual. Res. Psychol..

[19] Dyda A (2021). Managing the digital disruption associated with COVID-19-driven rapid digital transformation in Brisbane, Australia. Appl. Clin. Inform..

[20] Su J-Y, Guthridge S, He VY, Howard D, Leach AJ (2020). Impact of hearing impairment on early childhood development in Australian Aboriginal children: a data linkage study. J. Paediatr. Child Health.

[21] Sinclair G, Collins S, Arbour L, Vallance H (2019). The p.P479L variant in CPT1A is associated with infectious disease in a BC First Nation. Paediatr. Child Health.

[22] Pena-Sanchez JN (2022). Increasing PRevalence and Stable Incidence Rates of Inflammatory Bowel Disease among First Nations: Population-based Evidence from a Western Canadian Province. Inflamm. Bowel Dis..

[23] McInerney C (2019). Benefits of not smoking during pregnancy for Australian Aboriginal and Torres Strait Islander women and their babies: a retrospective cohort study using linked data. BMJ Open.

[24] Lima F, Shepherd C, Wong J, O’Donnell M, Marriott R (2019). Trends in mental health related contacts among mothers of Aboriginal children in Western Australia (1990-2013): a linked data population-based cohort study of over 40 000 children. BMJ Open.

[25] Leckning B (2021). Patterns of child protection service involvement by Aboriginal children associated with a higher risk of self-harm in adolescence: a retrospective population cohort study using linked administrative data. Child Abus. Negl..

[26] Lavoie JG (2018). Hospitalization for mental health related ambulatory care sensitive conditions: what are the trends for First Nations in British Columbia?. Int. J. Equity Health.

[27] Lavoie JG (2022). Kivalliq Inuit women travelling to Manitoba for birthing: findings from the Qanuinngitsiarutiksait study. BMC Pregnancy Childbirth.

[28] Kearns T (2013). Clinic attendances during the first 12 months of life for Aboriginal children in five remote communities of northern Australia. PloS ONE.

[29] Katzenellenbogen JM, Miller LJ, Somerford P, McEvoy S, Bessarab D (2015). Strategic information for hospital service planning: a linked data study to inform an urban Aboriginal Health Liaison Officer program in Western Australia. Aust. Health Rev..

[30] Hare MJL (2022). Prevalence and incidence of diabetes among Aboriginal people in remote communities of the Northern Territory, Australia: a retrospective, longitudinal data-linkage study. BMJ Open.

[31] Harasemiw O (2021). Impact of point-of-care screening for hypertension, diabetes and progression of chronic kidney disease in rural Manitoba Indigenous communities. Can. Med. Assoc. J..

[32] Frejuk KL (2021). Impact of a screen, triage and treat program for identifying chronic disease risk in Indigenous children. Can. Med. Assoc. J..

[33] Enns JE (2021). An unconditional prenatal income supplement is associated with improved birth and early childhood outcomes among First Nations children in Manitoba, Canada: a population-based cohort study. BMC Pregnancy Childbirth.

[34] Clark W (2022). Trends in Inuit health services utilisation in Manitoba: findings from the Qanuinngitsiarutiksait study. Int. J. Circumpolar Health.

[35] Carlin E (2022). Implementation of the ‘Kimberley Mum’s Mood Scale’ across primary health care services in the Kimberley region of Western Australia: a mixed methods assessment. PloS ONE.

[36] Bhat SK, Marriott R, Galbally M, Shepherd C (2020). Psychosocial disadvantage and residential remoteness is associated with Aboriginal women’s mental health prior to childbirth. Int. J. Popul. Data Sci..

[37] Singleton RJ (2022). Impact of a prenatal Vitamin D supplementation program on Vitamin D deficiency, rickets and early childhood caries in an alaska native population. Nutrients.

[38] Mitsch A, Surendera Babu A, Seneca D, Whiteside YO, Warne D (2017). HIV care and treatment of American Indians/Alaska natives with diagnosed HIV infection—27 states and the District of Columbia, 2012. Int. J. STD AIDS.

[39] Lillie KM, Shaw J, Jansen KJ, Garrison MM (2021). Buprenorphine/naloxone for opioid use disorder among Alaska Native and American Indian People. J. Addict. Med..

[40] Kelly L (2019). Prevalence of chronic kidney disease and cardiovascular comorbidities in adults in First Nations communities in northwest Ontario: a retrospective observational study. CMAJ Open.

[41] Franz C (2020). Community-based outreach associated with increased health utilization among Navajo individuals living with diabetes: a matched cohort study. BMC Health Serv. Res..

[42] Ferucci ED, Arnold RI, Holck P (2022). Factors associated with telemedicine use for chronic disease specialty care in the Alaska Tribal Health System, 2015-2019. Telemed. J. e-health.

[43] Ferucci, E. D., Arnold, R. I. & Holck, P. Health care utilization in Alaska Native people receiving chronic disease specialty care by videoconsultation compared to propensity-matched controls. *J. Telemed. Telecare*10.1177/1357633X221107999 (2022). 10.1177/1357633X221107999 PMC12178267 35733375

[44] Chi DL (2020). Supply of care by dental therapists and emergency dental consultations in Alaska native communities in the Yukon-Kuskokwim delta: a mixed methods evaluation. Commun. Dent. Health.

[45] Chi DL, Lenaker D, Mancl L, Dunbar M, Babb M (2018). Dental therapists linked to improved dental outcomes for Alaska Native communities in the Yukon-Kuskokwim Delta. J. Public Health Dent..

[46] Chan BTB, Sodhi SK, Mecredy GC, Farrell T, Gordon J (2021). Diabetes prevalence and complication rates: In individual First Nations communities in the Sioux Lookout region of Ontario. Can. Fam. Phys..

[47] Carlin E, Cox Z, Orazi K, Derry KL, Dudgeon P (2022). Exploring mental health presentations in remote Aboriginal Community Controlled Health Services in the Kimberley Region of Western Australia using an audit and file reviews. Int. J. Environ. Res. Public Health.

[48] Bradley C (2020). Establishment of a sentinel surveillance network for sexually transmissible infections and blood borne viruses in Aboriginal primary care services across Australia: the ATLAS project. BMC health Serv. Res..

[49] Thompson F (2022). Using health check data to understand risks for dementia and cognitive impairment among torres strait islander and aboriginal peoples in Northern Queensland—a data linkage study. Front. Public Health.

[50] Mendlowitz AB (2021). Healthcare costs associated with hepatitis C virus infection in the First Nations populations in Ontario…The Canadian Association for the Study of the Liver (CASL), the Canadian Network on Hepatitis C (CanHepC) and the ­Canadian Association of Hepatology Nurses (CAHN), Canadian Liver Meeting (Virtual), May 2-5, 2021. Can. Liver J..

[51] Lakhan P (2022). Challenges of conducting kidney health checks among patients at risk of chronic kidney disease and attending an urban Aboriginal and Torres Strait Islander primary healthcare service. Aust. J. Prim. Health.

[52] Hosking K (2020). Data linkage and computerised algorithmic coding to enhance individual clinical care for Aboriginal people living with chronic hepatitis B in the Northern Territory of Australia—is it feasible?. PloS ONE.

[53] Lasry O (2016). Traumatic brain injury in a rural indigenous population in Canada: a community-based approach to surveillance. CMAJ Open.

[54] Zhao Y, Wright J, Guthridge S, Lawton P (2013). The relationship between number of primary health care visits and hospitalisations: evidence from linked clinic and hospital data for remote Indigenous Australians. BMC Health Serv. Res..

[55] West C, Fitts MS, Rouen C, Muller R, Clough AR (2019). Cause and incidence of injuries experienced by children in remote Cape York Indigenous communities. Aust. J. Prim. Health.

[56] Struck S (2021). An unconditional prenatal cash benefit is associated with improved birth and early childhood outcomes for Metis families in Manitoba, Canada. Child. Youth Serv. Rev..

[57] Spaeth BA, Shephard MD, Schatz S (2014). Point-of-care testing for haemoglobin A1c in remote Australian Indigenous communities improves timeliness of diabetes care. Rural Remote Health.

[58] Schaefer KR (2019). Differences in service utilization at an urban tribal health organization before and after Alzheimer’s disease or related dementia diagnosis: a cohort study. Alzheimer’s Dement..

[59] Schaefer KR, Muller CJ, Smith JJ, Avey JP, Shaw JL (2022). Using the electronic health record to identify suicide risk factors in an Alaska Native Health System. Psychol. Serv..

[60] Ryan T (2021). Comparing health outcomes of rural and urban diabetes patients: an audit of a māori health provider. Kai Tiaki Nurs. Res..

[61] Muller CJ (2017). Text message reminders increased colorectal cancer screening in a randomized trial with Alaska Native and American Indian people. Cancer.

[62] Middleton J (2021). Temperature and place associations with Inuit mental health in the context of climate change. Environ. Res..

[63] Mera J (2020). Evaluation of the Cherokee Nation Hepatitis C Virus elimination program in the first 22 months of implementation. JAMA Netw. open.

[64] Mera J (2019). Retrospective study demonstrating high rates of sustained virologic response after treatment with direct-acting antivirals among American Indian/Alaskan Natives. Open Forum Infect. Dis..

[65] Howarth T (2020). Antibiotic use for Australian Aboriginal children in three remote Northern Territory communities. PloS ONE.

[66] Hla TK (2020). A “one stop liver shop” approach improves the cascade-of-care for Aboriginal and Torres Strait Islander Australians living with chronic hepatitis B in the Northern Territory of Australia: results of a novel care delivery model. Int. J. Equity Health.

[67] Gu Y, Warren J, Walker N, Kennelly J (2013). Gender differences in cardiovascular disease risk management for Pacific Islanders in primary care. Qual. Prim. Care.

[68] Gardner S (2016). Picture of the health status of Aboriginal children living in an urban setting of Sydney. Aust. Health Rev..

[69] Freeman J (2018). Can a child and family health service improve early childhood health outcomes in an urban Aboriginal community?. J. Paediatr. Child Health.

[70] Coughlin R, Kushman E, Copeland G, Wilson M (2013). Pregnancy and birth outcome improvements for american indians in the healthy start project of the inter-tribal council of Michigan, 1998-2008. Matern. Child Health J..

[71] Campbell S (2019). Childhood infection, antibiotic exposure and subsequent metabolic risk in adolescent and young adult Aboriginal Australians: practical implications. Aust. J. Prim. Health.

[72] Askew DA, Jennings WJ, Hayman NE, Schluter PJ, Spurling GK (2019). Knowing our patients: a cross-sectional study of adult patients attending an urban Aboriginal and Torres Strait Islander primary healthcare service. Aust. J. Prim. Health.

[73] Smylie J, Firestone M, Spiller MW (2018). Our health counts: population-based measures of urban Inuit health determinants, health status, and health care access. Can. J. Public Health.

[74] Mamakwa S (2017). Evaluation of 6 remote First Nations community-based buprenorphine programs in northwestern Ontario: retrospective study. Can. Fam. Phys..

[75] Li M, McDermott R (2016). High absolute risk of severe infections among Indigenous adults in rural northern Australia is amplified by diabetes—a 7 year follow up study. J. Diabetes Complic..

[76] Li M, McDermott R (2015). Smoking, poor nutrition, and sexually transmitted infections associated with pelvic inflammatory disease in remote North Queensland Indigenous communities, 1998-2005. BMC Women’s Health.

[77] Campbell SK, Lynch J, Esterman A, McDermott R (2013). Pre-pregnancy predictors of hypertension in pregnancy among Aboriginal and Torres Strait Islander women in north Queensland, Australia; a prospective cohort study. BMC Public Health.

[78] Daws K (2014). Implementing a working together model for Aboriginal patients with acute coronary syndrome: an Aboriginal Hospital Liaison Officer and a specialist cardiac nurse working together to improve hospital care. Aust. Health Rev..

[79] Best LG (2017). Genetic variants and risk of asthma in an American Indian population. Ann. Allergy, Asthma Immunol..

[80] Griffiths EK, Marley JV, Friello D, Atkinson DN (2016). Uptake of long-acting, reversible contraception in three remote aboriginal communities: a population-based study. Med. J. Aust..

[81] Shmerling E, Creati M, Belfrage M, Hedges S (2020). The health needs of Aboriginal and Torres Strait Islander children in out-of-home care. J. Paediatr. Child Health.

[82] Goins RT, Noonan C, Winchester B, Brock D (2019). Depressive symptoms and all-cause mortality in older American Indians with type 2 diabetes mellitus. J. Am. Geriatr. Soc..

[83] Bruden DJT (2015). Eighteen years of respiratory syncytial virus surveillance: changes in seasonality and hospitalization rates in Southwestern Alaska Native children. Pediatr. Infect. Dis. J..

[84] Shaw JL (2022). Validating a predictive algorithm for suicide risk with Alaska Native populations. Suicide Life-Threat. Behav..

[85] Manifold A (2019). Complex diabetes screening guidelines for high-risk adolescent Aboriginal Australians: a barrier to implementation in primary health care. Aust. J. Prim. Health.

[86] Khodra B, Stevens AM, Ferucci ED (2020). Prevalence of Juvenile Idiopathic Arthritis in the Alaska Native Population. Arthritis Care Res..

[87] Keck JW (2014). Influenza surveillance using electronic health records in the American Indian and Alaska Native population. J. Am. Med. Inform. Assoc..

[88] Hu J, Basit T, Nelson A, Crawford E, Turner L (2019). Does attending Work It Out—a chronic disease self-management program—affect the use of other health services by urban Aboriginal and Torres Strait Islander people with or at risk of chronic disease? A comparison between program participants and non-participants. Aust. J. Prim. health.

[89] Denise ST, Joy LJ, Annette JB, Sam S (2013). Maternal-Infant Health Outcomes and Nursing Practice in a Remote First Nations Community in Northern Canada. Can. J. Nurs. Res..

[90] Le-Morawa, N. et al. Effectiveness of a COVID-19 vaccine rollout in a highly affected American Indian Community, San Carlos Apache Tribe, December 2020–February 2021. *Public Health Reports* (Washington, D.C.: 1974) 10.1177/00333549221120238 (2022). 10.1177/00333549221120238 PMC10515982 36017554

[91] Hoy WE, Swanson CE, Hope A, Smith J, Masters C (2014). Evidence for improved patient management through electronic patient records at a Central Australian Aboriginal Health Service. Aust. N.Z. J. Public Health.

[92] Gordon J (2015). Acute rheumatic fever in first nations communities in northwestern Ontario: social determinants of health “bite the heart”. Can. Fam. Phys..

[93] Goins RT, Noonan C, Gonzales K, Winchester B, Bradley VL (2017). Association of depressive symptomology and psychological trauma with diabetes control among older American Indian women: does social support matter?. J. Diabetes Complic..

[94] Taylor MM (2013). Use of expedited partner therapy among chlamydia cases diagnosed at an urban Indian health centre, Arizona. Int. J. STD AIDS.

[95] Gibberd AJ, Simpson JM, McNamara BJ, Eades SJ (2019). Maternal fetal programming of birthweight among Australian Aboriginal infants: a population-based data linkage study. Lancet Glob. Health.

[96] Takashima M, Lambert SB, Paynter S, Ware RS (2019). Relative effectiveness of revaccination with 23-valent pneumococcal polysaccharide vaccine in preventing invasive pneumococcal disease in adult Aboriginal and Torres Strait Islander people, Australia. Vaccine.

[97] Campbell M (2022). Health care cost of crusted scabies in Aboriginal communities in the Northern Territory, Australia. PLoS Negl. Trop. Dis..

[98] Tran-Duy A (2020). Development and use of prediction models for classification of cardiovascular risk of remote indigenous Australians. Heart Lung Circ..

[99] Reilley B (2018). Assessing new diagnoses of HIV among American Indian/Alaska Natives served by the Indian Health Service, 2005-2014. Public Health Rep..

[100] Davis B, McLean A, Sinha AK, Falhammar H (2013). A threefold increase in gestational diabetes over two years: Review of screening practices and pregnancy outcomes in Indigenous women of Cape York, Australia. Aust. N.Z. J. Obstet. Gynaecol..

[101] Nicholls SG (2015). The REporting of Studies Conducted Using Observational Routinely-Collected Health Data (RECORD) statement: methods for arriving at consensus and developing reporting guidelines. PLoS ONE.

[102] Kowal E, Llamas B, Tishkoff S (2017). Data-sharing for Indigenous Peoples. Nature.

[103] Simon de L, Harshana L, Concetta Tania Di I, Tom C, Siaw-Teng L (2015). Using routinely collected health data for surveillance, quality improvement and research: Framework and key questions to assess ethics and privacy and enable data access. BMJ Health Care Inform..

[104] Lovett R, et al. Good data practices for indigenous data sovereignty and governance. In: *Good Data* (eds Daly, A., Devitt S.K., & Mann, M.) 26–36 (Institute of Network Cultures, Amsterdam, 2019).

[105] Love RP (2022). Developing Data Governance Agreements with Indigenous Communities in Canada: toward equitable tuberculosis programming, research, and reconciliation. Health Hum. Rights.

