# Supplementary material for: Indigenous data governance approaches applied in research using routinely collected health data: a scoping review
Source: NPJ Digit Med. 2024 Mar 15;7:68. doi: 10.1038/s41746-024-01070-3 (PMC10943072; doi:10.1038/s41746-024-01070-3)
Supplement: Supplementary file 1 — Supplementary Tables [file 41746_2024_1070_MOESM1_ESM.pdf]

Supplementary Table 1 - PRISMA Checklist

Supplemental Material 1. Preferred Reporting Items for Systematic reviews and Meta-Analyses extension for Scoping Reviews (PRISMA-ScR) Checklist

SECTION	ITEM	PRISMA-ScR CHECKLIST ITEM	REPORTED ON PAGE #
**TITLE**			
Title	1	Identify the report as a scoping review.	Page 1
**ABSTRACT**			
Structured summary	2	Provide a structured summary that includes (as applicable): background, objectives, eligibility criteria, sources of evidence, charting methods, results, and conclusions that relate to the review questions and objectives.	Page 3
**INTRODUCTION**			
Rationale	3	Describe the rationale for the review in the context of what is already known. Explain why the review questions/objectives lend themselves to a scoping review approach.	Page 4
Objectives	4	Provide an explicit statement of the questions and objectives being addressed with reference to their key elements (e.g., population or participants, concepts, and context) or other relevant key elements used to conceptualize the review questions and/or objectives.	Page 5
**METHODS**			
Protocol and registration	5	Indicate whether a review protocol exists; state if and where it can be accessed (e.g., a Web address); and if available, provide registration information, including the registration number.	N/A
Eligibility criteria	6	Specify characteristics of the sources of evidence used as eligibility criteria (e.g., years considered, language, and publication status), and provide a rationale.	Page 6
Information sources*	7	Describe all information sources in the search (e.g., databases with dates of coverage and contact with authors to identify additional sources), as well as the date the most recent search was executed.	Page 5
Search	8	Present the full electronic search strategy for at least 1 database, including any limits used, such that it could be repeated.	Supplementary Table 2
Selection of sources of evidence†	9	State the process for selecting sources of evidence (i.e., screening and eligibility) included in the scoping review.	Page 6
Data charting process‡	10	Describe the methods of charting data from the included sources of evidence (e.g., calibrated forms or forms that have been tested by the team before their use, and whether data charting was done independently or in duplicate) and any processes for obtaining and confirming data from investigators.	Page 7
Data items	11	List and define all variables for which data were sought and any assumptions and simplifications made.	Page 6
Critical appraisal of individual sources of evidence§	12	If done, provide a rationale for conducting a critical appraisal of included sources of evidence; describe the methods used and how this information was used in any data synthesis (if appropriate).	N/A
Synthesis of results	13	Describe the methods of handling and summarizing the data that were charted.	N/A

SECTION	ITEM	PRISMA-ScR CHECKLIST ITEM	REPORTED ON PAGE #
**RESULTS**			
Selection of sources of evidence	14	Give numbers of sources of evidence screened, assessed for eligibility, and included in the review, with reasons for exclusions at each stage, ideally using a flow diagram.	Page 6, Figure 1
Characteristics of sources of evidence	15	For each source of evidence, present characteristics for which data were charted and provide the citations.	Page 6-7, Table 2
Critical appraisal within sources of evidence	16	If done, present data on critical appraisal of included sources of evidence (see item 12).	N/A
Results of individual sources of evidence	17	For each included source of evidence, present the relevant data that were charted that relate to the review questions and objectives.	Page 7 -13
Synthesis of results	18	Summarize and/or present the charting results as they relate to the review questions and objectives.	Page 7 -13
**DISCUSSION**			
Summary of evidence	19	Summarize the main results (including an overview of concepts, themes, and types of evidence available), link to the review questions and objectives, and consider the relevance to key groups.	Page 13-14
Limitations	20	Discuss the limitations of the scoping review process.	Page 14
Conclusions	21	Provide a general interpretation of the results with respect to the review questions and objectives, as well as potential implications and/or next steps.	Page 14
**FUNDING**			
Funding	22	Describe sources of funding for the included sources of evidence, as well as sources of funding for the scoping review. Describe the role of the funders of the scoping review.	Page 14

JBI = Joanna Briggs Institute; PRISMA-ScR = Preferred Reporting Items for Systematic reviews and Meta-Analyses extension for Scoping Reviews.
* Where *sources of evidence* (see second footnote) are compiled from, such as bibliographic databases, social media platforms, and Web sites.
† A more inclusive/heterogeneous term used to account for the different types of evidence or data sources (e.g., quantitative and/or qualitative research, expert opinion, and policy documents) that may be eligible in a scoping review as opposed to only studies. This is not to be confused with *information sources* (see first footnote).
‡ The frameworks by Arksey and O'Malley (6) and Levac and colleagues (7) and the JBI guidance (4, 5) refer to the process of data extraction in a scoping review as data charting.
§ The process of systematically examining research evidence to assess its validity, results, and relevance before using it to inform a decision. This term is used for items 12 and 19 instead of "risk of bias" (which is more applicable to systematic reviews of interventions) to include and acknowledge the various sources of evidence that may be used in a scoping review (e.g., quantitative and/or qualitative research, expert opinion, and policy document).

From: Tricco AC, Lillie E, Zarin W, O'Brien KK, Colquhoun H, Levac D, et al. PRISMA Extension for Scoping Reviews (PRISMAScR): Checklist and Explanation. Ann Intern Med. 2018;169:467–473. doi: 10.7326/M18-0850.

Supplementary Table 2 - Search Strategy

Database	Search term	Date run	Total Results	Last 10 Years Results
Medline OVID	(exp *"Health Services, Indigenous"/ OR exp *"Indigenous Peoples"/ OR exp *"American Native Continental Ancestry Group"/ OR exp *"Native Hawaiian or Other Pacific Islander"/ OR exp *"United States Indian Health Service" OR indigenous*.ti. OR aborigin*.ti. OR "torres strait islander*".ti. OR "first nation".ti. OR "first nations".ti. OR metis.ti. OR "alaska native*".ti. OR "alaskan native*".ti. OR "american indian*".ti. OR maori*.ti. OR sami*.ti. OR saami*.ti. OR inuit*.ti. OR tribe*.ti. OR tribal*.ti. OR "native Hawaii*".ti OR Cherokee.ti OR Navajo.ti)<br>AND<br>("data link*".tw. OR "linked data".tw. OR "record link*".tw. OR "linked record*".tw. OR ((electronic*.tw. OR digital*.tw. OR computer*.tw.) ADJ2 (health record*.tw. OR medical record*.tw. OR patient record*.tw.)) OR exp "Medical Records Systems, Computerized"/ OR exp "Medical Record Linkage"/ OR exp "Routinely Collected Health Data"/ OR "Routinely Collected Health Data".tw. OR "administrative data".tw. OR "administrative health data".tw.)	6-Dec-22	445	331
EMBASE	('indigenous people'/exp/mj OR 'canadian aboriginal'/exp/mj OR 'native hawaiian'/exp/mj OR 'oceanic ancestry group'/exp/mj OR 'Australian Aborigine'/exp/mj OR 'indigen*':ti OR aborigin*:ti OR 'torres strait island*':ti OR 'first nation':ti OR 'first nations':ti OR metis:ti OR 'alaskan native':ti OR 'alaska native':ti OR 'american indian':ti OR 'maori':ti OR 'sami':ti OR 'saami':ti OR inuit:ti OR Cherokee:ti OR Navajo:ti)<br>AND<br>('data linkage'/exp OR 'data link*':ab,ti,kw OR 'linked data':ab,ti,kw OR 'record link*':ab,ti,kw OR 'linked record*':ab,ti,kw OR 'electronic health record'/exp OR 'electronic medical record'/exp OR 'electronic medical record system'/exp OR 'electronic patient record'/exp OR ((electronic* OR digital* OR computer*) NEAR/2 ('health record*' OR 'medical record*' OR 'patient record*'))<br>OR 'administrative health data':ab,ti,kw OR 'administrative data':ab,ti,kw OR ' Routinely Collected Health Data ':ab,ti,kw OR 'administrative health data'/exp OR 'routinely collected health data'/exp)	6-Dec-22	508	398
CINAHL	((MM "Health Services, Indigenous") OR (MM "Indigenous Peoples+") OR (MM "Indigenous Health") OR TI ('indigen*' OR aborigin* OR 'torres strait island*' OR 'first nation' OR 'first nations' OR metis OR 'alaskan native' OR 'alaska native' OR 'american indian' OR 'maori' OR 'sami' OR saami OR inuit OR Cherokee OR Navajo))<br>AND<br>((MH "Health and Disease") OR (MH "Health Services Administration") OR AB (health* OR mortal* OR morbid* OR hospi* OR medic*) OR TI (health* OR mortal* OR morbid* OR hospi* OR medic*))<br>AND<br>((MH "Routinely Collected Health Data") OR (MH "Clinical Data Repository") OR (MH "Electronic Health Records") OR (MH "Medical Records") OR (MH "Medical Record Linkage")<br>OR ((electronic* OR digital* OR computer*) N2 ("'health record*'" OR "'medical record*'" OR "'patient record*'"))<br>OR AB ('administrative data' OR 'administrative health data' OR 'Routinely Collected Health Data' OR 'data link*' OR 'linked data' OR 'record link*' OR 'linked record*') OR TI ('administrative data' OR 'administrative health data' OR 'Routinely Collected Health Data' OR 'data link*' OR 'linked data' OR 'record link*' OR 'linked record*'))	6-Dec-22	376	260
Web of Science	((TI=("Indigenous Peoples" OR "Indigenous Canadians" OR "American Native" OR "Native Hawaiian" OR "Pacific Islander" OR "indigenous*" OR "aborigin*" OR "torres strait islander" OR "first nation" OR "first nations" OR "metis" OR "alaska native*" OR "alaskan native*" OR "american Indian" OR "maori*" OR "sami*" OR "saami*" OR "inuit*" OR "tribe*" OR "tribal*" OR "Cherokee" OR "Navajo")) OR (AK=("Indigenous Peoples" OR "Indigenous Canadians" OR "American Native" OR "Native Hawaiian" OR "Pacific Islander" OR "indigenous*" OR "aborigin*" OR "torres strait islander" OR "first nation"	6-Dec-22	473	377

	OR “first nations” OR “metis” OR “alaska native*” OR “alaskan native*” OR “american Indian” OR “maori*” OR “sami*” OR “saami*” OR “inuit*” OR “tribe*” OR “tribal*” OR “Cherokee” OR “Navajo”)) OR (KP=(“Indigenous Peoples” OR “Indigenous Canadians” OR “American Native” OR “Native Hawaiian” OR “Pacific Islander” OR “indigenous*” OR “aborigin*” OR “torres strait islander” OR “first nation” OR “first nations” OR “metis” OR “alaska native*” OR “alaskan native*” OR “american Indian” OR “maori*” OR “sami*” OR “saami*” OR “inuit*” OR “tribe*” OR “tribal*” OR “Cherokee” OR “Navajo”)))<br>AND<br>(TS=("data link*" OR "linked data" OR ((electronic* OR digital* OR computer*) NEAR/2 ("health record*" OR "medical record*" OR "personal record*" OR "patient record*")) OR "Medical Records Systems, Computerized" OR "Medical Record Linkage" OR "Routinely Collected Health Data" OR "administrative data" OR "administrative health data" OR "record link*" OR "linked record*")<br>AND<br>TS=("Health*" OR "mortal*" OR "morbid*" OR "hospi*" OR "medic*" OR "Diseas*" OR "sick*" OR "Psych*" or "surg*" OR "treat*" OR "care*"))			
ATSIHealth	(Title: "data link*" OR "linked data" OR ((electronic* OR digital* OR computer*) AND ("health record*" OR "medical record*" OR "personal record*" OR "patient record*")) OR "Medical Records Systems, Computerized" OR "Medical Record Linkage" OR "Routinely Collected Health Data" OR "administrative data" OR "administrative health data" OR "record link*" OR "linked record*")<br>OR<br>(Abstract: "data link*" OR "linked data" OR ((electronic* OR digital* OR computer*) AND ("health record*" OR "medical record*" OR "personal record*" OR "patient record*")) OR "Medical Records Systems, Computerized" OR "Medical Record Linkage" OR "Routinely Collected Health Data" OR "administrative data" OR "administrative health data" OR "record link*" OR "linked record*")	6-Dec-22	39	23
**Combined**				**1,012**
**Combined de-duped in EndNote**				**662**
**Combined de-duped in Covidence**				**580**

Supplementary Table 3 - Data Extraction

#	Lead author surname	Year	Title	Country	Region study was conducted	Indigenous group/s	Gender	Outcome measured	Number of participants
1	Zhao	2013	The relationship between number of primary health care visits and hospitalisations: evidence from linked clinic and hospital data for remote Indigenous Australians	Australia	Rural	Aboriginal Australian; Torres Strait Islander	All	Healthcare Utilisation and Access	52,739
2	West	2019	Cause and incidence of injuries experienced by children in remote Cape York Indigenous communities	Australia	Rural	Aboriginal Australian; Torres Strait Islander	All	Other Health Conditions	563
3	Tran-Duy	2020	Development and Use of Prediction Models for Classification of Cardiovascular Risk of Remote Indigenous Australians	Australia	Rural	Aboriginal Australian; Torres Strait Islander	All	Chronic Diseases and Comorbidities	1,583
4	Thompson	2022	Using Health Check Data to Understand Risks for Dementia and Cognitive Impairment Among Torres Strait Islander and Aboriginal Peoples in Northern Queensland-A Data Linkage Study	Australia	Rural	Aboriginal Australian; Torres Strait Islander	All	Chronic Diseases and Comorbidities	64
5	Taylor	2013	Use of expedited partner therapy among chlamydia cases diagnosed at an urban Indian health centre, Arizona	United States	Urban	Alaska Native; American Indian	All	Healthcare Utilisation and Access	492
6	Takashima	2019	Relative effectiveness of revaccination with 23-valent pneumococcal polysaccharide vaccine in preventing invasive pneumococcal disease in adult Aboriginal and Torres Strait Islander people, Australia	Australia	Urban and Rural	Aboriginal Australian; Torres Strait Islander	All	Infectious Diseases	12,809
7	Su	2020	Impact of hearing impairment on early childhood development in Australian Aboriginal children: A data linkage study	Australia	Rural	Aboriginal Australian	All	Maternal and Child Health	1,037
8	Struck	2021	An unconditional prenatal cash benefit is associated with improved birth and early childhood outcomes for Metis families in Manitoba, Canada	Canada	Urban and Rural	MÈtis	Female	Maternal and Child Health	4,891
9	Stokes	2018	Multimorbidity in Maori and Pacific patients: cross-sectional study in a Dunedin general practice	New Zealand	Urban	M?ori; Pacific Islander	All	Chronic Diseases and Comorbidities	232
10	Spaeth	2014	Point-of-care testing for haemoglobin A1c in remote Australian Indigenous communities improves timeliness of diabetes care	Australia	Rural	Aboriginal Australian; Torres Strait Islander	All	Healthcare Utilisation and Access	907
11	Smylie	2018	Our health counts: population-based measures of urban Inuit health determinants, health status, and health care access	Canada	Urban	Inuit	All	Healthcare Utilisation and Access	341
12	Singleton	2022	Impact of a Prenatal Vitamin D Supplementation Program on Vitamin D Deficiency, Rickets and Early Childhood Caries in an Alaska Native Population	United States	Rural	Alaska Native	All	Maternal and Child Health	1,522

13	Sinclair	2019	The p.P479L variant in CPT1A is associated with infectious disease in a BC First Nation	Canada	Not described	First Nations living in Canada	All	Infectious Diseases	150
14	Shmerling	2020	The health needs of Aboriginal and Torres Strait Islander children in out-of-home care	Australia	Urban	Aboriginal Australian; Torres Strait Islander	All	Maternal and Child Health	103
15	Shaw	2022	Validating a predictive algorithm for suicide risk with Alaska Native populations	United States	Not described	Alaska Native; American Indian	All	Mental Health and Suicide Risk	10,864
16	Schaefer	2019	Differences in service utilization at an urban tribal health organization before and after Alzheimer's disease or related dementia diagnosis: A cohort study	United States	Urban and Rural	Alaska Native; American Indian	All	Healthcare Utilisation and Access	1,591
17	Schaefer	2022	Using the electronic health record to identify suicide risk factors in an Alaska Native Health System	United States	Urban and Rural	Alaska Native; American Indian	All	Mental Health and Suicide Risk	1,483
18	Ryan	2021	COMPARING HEALTH OUTCOMES OF RURAL AND URBAN DIABETES PATIENTS: AN AUDIT OF A MƒÄORI HEALTH PROVIDER	New Zealand	Urban and Rural	M?ori	All	Healthcare Utilisation and Access	372
19	Reilley	2018	Assessing New Diagnoses of HIV Among American Indian/Alaska Natives Served by the Indian Health Service, 2005-2014	United States	Rural	Alaska Native; American Indian	All	Infectious Diseases	2,273
20	Ray	2020	Health care utilisation changes among Alaska Native adults after participation in an indigenous community programme to address adverse life experiences: a propensity score-matched analysis	United States	Urban and Rural	Alaska Native; American Indian	All	Healthcare Utilisation and Access	180
21	Pe√±a-S√°nchez	2022	Increasing Prevalence and Stable Incidence Rates of Inflammatory Bowel Disease Among First Nations: Population-Based Evidence From a Western Canadian Province	Canada	Urban and Rural	First Nations living in Canada	All	Chronic Diseases and Comorbidities	140
22	Muller	2017	Text message reminders increased colorectal cancer screening in a randomized trial with Alaska Native and American Indian people	United States	Urban and Rural	Alaska Native; American Indian	All	Healthcare Utilisation and Access	2,386
23	Mitsch	2017	HIV care and treatment of American Indians/Alaska natives with diagnosed HIV infection - 27 states and the District of Columbia, 2012	United States	Urban and Rural	Alaska Native; American Indian	All	Healthcare Utilisation and Access	58
24	Middleton	2021	Temperature and place associations with Inuit mental health in the context of climate change	Canada	Rural	Inuit	All	Mental Health and Suicide Risk	5,373
25	Mera	2020	Evaluation of the Cherokee Nation Hepatitis C Virus Elimination Program in the First 22 Months of Implementation	United States	Rural	Alaska Native; American Indian	All	Infectious Diseases	74,039
26	Mera	2019	Retrospective Study Demonstrating High Rates of Sustained Virologic Response after Treatment with Direct-Acting Antivirals among American Indian/Alaskan Natives	United States	Urban and Rural	Alaska Native; American Indian	All	Infectious Diseases	280

27	Mendlowitz	2021	Healthcare costs associated with hepatitis C virus infection in the First Nations populations in Ontario...The Canadian Association for the Study of the Liver (CASL), the Canadian Network on Hepatitis C (CanHepC) and the ¬≠Canadian Association of Hepatology Nurses (CAHN), Canadian Liver Meeting (Virtual), May 2-5, 2021	Canada	Urban and Rural	First Nations living in Canada	All	Infectious Diseases	6,012
28	McInerney	2019	Benefits of not smoking during pregnancy for Australian Aboriginal and Torres Strait Islander women and their babies: a retrospective cohort study using linked data	Australia	Urban and Rural	Aboriginal Australian; Torres Strait Islander	All	Maternal and Child Health	31,631
29	Manifold	2019	Complex diabetes screening guidelines for high-risk adolescent Aboriginal Australians: a barrier to implementation in primary health care	Australia	Rural	Aboriginal Australian; Torres Strait Islander	All	Chronic Diseases and Comorbidities	269
30	Mamakwa	2017	Evaluation of 6 remote First Nations community-based buprenorphine programs in northwestern Ontario: Retrospective study	Canada	Rural	First Nations living in Canada	All	Other Health Conditions	526
31	Lima	2019	Trends in mental health related contacts among mothers of Aboriginal children in Western Australia (1990-2013): a linked data population-based cohort study of over 40 000 children	Australia	Urban and Rural	Aboriginal Australian	All	Maternal and Child Health; Mental Health and Suicide Risk; Healthcare Utilisation and Access	43,383
32	Lillie	2021	Buprenorphine/Naloxone for Opioid Use Disorder Among Alaska Native and American Indian People	United States	Urban	Alaska Native; American Indian	All	Other Health Conditions	240
33	Li	2016	High absolute risk of severe infections among Indigenous adults in rural northern Australia is amplified by diabetes - A 7 year follow up study	Australia	Rural	Aboriginal Australian; Torres Strait Islander	All	Public Health and Prevention	2,787
34	Li	2015	Smoking, poor nutrition, and sexually transmitted infections associated with pelvic inflammatory disease in remote North Queensland Indigenous communities, 1998-2005	Australia	Rural	Aboriginal Australian; Torres Strait Islander	Female	Infectious Diseases	1,445
35	Leckning	2021	Patterns of child protection service involvement by Aboriginal children associated with a higher risk of self-harm in adolescence: A retrospective population cohort study using linked administrative data	Australia	Urban and Rural	Aboriginal Australian	All	Mental Health and Suicide Risk	6,476
36	Le-Morawa	2022	Effectiveness of a COVID-19 Vaccine Rollout in a Highly Affected American Indian Community, San Carlos Apache Tribe, December 2020-February 2021	United States	Rural	American Indian	All	Public Health and Prevention	16,178

37	Lavoie	2018	Hospitalization for mental health related ambulatory care sensitive conditions: what are the trends for First Nations in British Columbia?	Canada	Urban and Rural	First Nations living in Canada	All	Mental Health and Suicide Risk; Healthcare Utilisation and Access	96,808
38	Lavoie	2022	Kivalliq Inuit women travelling to Manitoba for birthing: findings from the Qanuinngitsiarutiksait study	Canada	Rural	Inuit	All	Healthcare Utilisation and Access	232
39	Lasry	2016	Traumatic brain injury in a rural indigenous population in Canada: a community-based approach to surveillance	Canada	Rural	First Nations living in Canada	All	Other Health Conditions	172
40	Lakhan	2022	Challenges of conducting kidney health checks among patients at risk of chronic kidney disease and attending an urban Aboriginal and Torres Strait Islander primary healthcare service	Australia	Urban	Aboriginal Australian; Torres Strait Islander	All	Chronic Diseases and Comorbidities	1,181
41	Khodra	2020	Prevalence of Juvenile Idiopathic Arthritis in the Alaska Native Population	United States	Urban and Rural	Alaska Native	All	Maternal and Child Health	42
42	Kelly	2019	Prevalence of chronic kidney disease and cardiovascular comorbidities in adults in First Nations communities in northwest Ontario: a retrospective observational study	Canada	Rural	First Nations living in Canada	All	Chronic Diseases and Comorbidities	16,170
43	Keck	2014	Influenza surveillance using electronic health records in the American Indian and Alaska Native population	United States	Urban and Rural	Alaska Native; American Indian	All	Public Health and Prevention	269
44	Kearns	2013	Clinic attendances during the first 12 months of life for Aboriginal children in five remote communities of northern Australia	Australia	Rural	Aboriginal Australian	All	Healthcare Utilisation and Access	320
45	Katzenellen bogen	2015	Strategic information for hospital service planning: a linked data study to inform an urban Aboriginal Health Liaison Officer program in Western Australia	Australia	Urban	Aboriginal Australian	All	Healthcare Utilisation and Access	5,113
46	Hu	2019	Does attending Work It Out ,Äì a chronic disease self-management program ,Äì affect the use of other health services by urban Aboriginal and Torres Strait Islander people with or at risk of chronic disease? A comparison between program participants and non-participants	Australia	Urban	Aboriginal Australian; Torres Strait Islander	All	Healthcare Utilisation and Access	815
47	Hoy	2014	Evidence for improved patient management through electronic patient records at a Central Australian Aboriginal Health Service	Australia	Rural	Aboriginal Australian	All	Chronic Diseases and Comorbidities	1,251
48	Howarth	2020	Antibiotic use for Australian Aboriginal children in three remote Northern Territory communities	Australia	Rural	Aboriginal Australian; Torres Strait Islander	All	Healthcare Utilisation and Access	124

49	Hosking	2020	Data linkage and computerised algorithmic coding to enhance individual clinical care for Aboriginal people living with chronic hepatitis B in the Northern Territory of Australia - Is it feasible?	Australia	Rural	Aboriginal Australian	All	Infectious Diseases	19,314
50	Hla	2020	A "one stop liver shop" approach improves the cascade-of-care for Aboriginal and Torres Strait Islander Australians living with chronic hepatitis B in the Northern Territory of Australia: results of a novel care delivery model	Australia	Rural	Aboriginal Australian; Torres Strait Islander	All	Infectious Diseases	83
51	Hare	2022	Prevalence and incidence of diabetes among Aboriginal people in remote communities of the Northern Territory, Australia: a retrospective, longitudinal data-linkage study	Australia	Rural	Aboriginal Australian	All	Chronic Diseases and Comorbidities	21,267
52	Harasemiw	2021	Impact of point-of-care screening for hypertension, diabetes and progression of chronic kidney disease in rural Manitoba Indigenous communities	Canada	Rural	First Nations living in Canada	All	Chronic Diseases and Comorbidities	1,353
53	Gu	2013	Gender differences in cardiovascular disease risk management for Pacific Islanders in primary care	New Zealand	Urban and Rural	Pacific Islander	All	Chronic Diseases and Comorbidities	10,863
54	Griffiths	2016	Uptake of long-acting, reversible contraception in three remote aboriginal communities: A population-based study	Australia	Rural	Aboriginal Australian; Torres Strait Islander	Female	Maternal and Child Health	566
55	Gordon	2015	Acute rheumatic fever in first nations communities in northwestern Ontario: Social determinants of health "bite the heart"	Canada	Rural	First Nations living in Canada	All	Chronic Diseases and Comorbidities	8
56	Goins	2019	Depressive Symptoms and All-Cause Mortality in Older American Indians with Type 2 Diabetes Mellitus	United States	Rural	American Indian	All	Chronic Diseases and Comorbidities	222
57	Goins	2017	Association of depressive symptomology and psychological trauma with diabetes control among older American Indian women: Does social support matter?	United States	Rural	American Indian	Female	Chronic Diseases and Comorbidities	81
58	Gibberd	2019	Maternal fetal programming of birthweight among Australian Aboriginal infants: a population-based data linkage study	Australia	Not described	Aboriginal Australian	All	Maternal and Child Health	20,978
59	Gardner	2016	Picture of the health status of Aboriginal children living in an urban setting of Sydney	Australia	Urban	Aboriginal Australian	All	Public Health and Prevention	205
60	Frejuk	2021	Impact of a screen, triage and treat program for identifying chronic disease risk in Indigenous children	Canada	Rural	First Nations living in Canada	All	Healthcare Utilisation and Access	324
61	Freeman	2018	Can a child and family health service improve early childhood health outcomes in an urban Aboriginal community?	Australia	Urban	Aboriginal Australian	All	Maternal and Child Health	639

62	Franz	2020	Community-based outreach associated with increased health utilization among Navajo individuals living with diabetes: a matched cohort study	United States	Rural	American Indian	All	Healthcare Utilisation and Access	3,053
63	Ferucci	2022	Factors Associated with Telemedicine Use for Chronic Disease Specialty Care in the Alaska Tribal Health System, 2015-2019	United States	Not described	Alaska Native	All	Healthcare Utilisation and Access	3,075
64	Ferucci	2022	Health care utilization in Alaska Native people receiving chronic disease specialty care by videoconsultation compared to propensity-matched controls	United States	Rural	Alaska Native	All	Healthcare Utilisation and Access	3,075
65	Enns	2021	An unconditional prenatal income supplement is associated with improved birth and early childhood outcomes among First Nations children in Manitoba, Canada: a population-based cohort study	Canada	Urban and Rural	First Nations living in Canada	Female	Maternal and Child Health	8,209
66	Ehmann	2013	Vogt-Koyanagi-Harada disease in First Nations and Metis of Northern Alberta	Canada	Rural	First Nations living in Canada; MÈtis	All	Other Health Conditions	19
67	Denise	2013	Maternal-Infant Health Outcomes and Nursing Practice in a Remote First Nations Community in Northern Canada	Canada	Rural	First Nations living in Canada	All	Maternal and Child Health	128
68	Daws	2014	Implementing a working together model for Aboriginal patients with acute coronary syndrome: an Aboriginal Hospital Liaison Officer and a specialist cardiac nurse working together to improve hospital care	Australia	Urban	Aboriginal Australian; Torres Strait Islander	All	Healthcare Utilisation and Access	15
69	Davis	2013	A threefold increase in gestational diabetes over two years: Review of screening practices and pregnancy outcomes in Indigenous women of Cape York, Australia	Australia	Rural	Aboriginal Australian; Torres Strait Islander	Female	Maternal and Child Health	261
70	Coughlin	2013	Pregnancy and Birth Outcome Improvements for American Indians in the Healthy Start Project of the Inter-Tribal Council of Michigan, 1998-2008	United States	Urban and Rural	American Indian	All	Maternal and Child Health	4,149
71	Clark	2022	Trends in Inuit health services utilisation in Manitoba: findings from the Qanuinngitsiarutiksait study	Canada	Urban and Rural	Inuit	All	Healthcare Utilisation and Access	11,430
72	Chi	2020	Supply of care by dental therapists and emergency dental consultations in Alaska native communities in the Yukon-Kuskokwim delta: a mixed methods evaluation	United States	Rural	Alaska Native	All	Dental Health	138,551
73	Chi	2018	Dental therapists linked to improved dental outcomes for Alaska Native communities in the Yukon-Kuskokwim Delta	United States	Rural	Alaska Native	All	Dental Health	28,191

74	Chan	2021	Diabetes prevalence and complication rates: In individual First Nations communities in the Sioux Lookout region of Ontario	Canada	Rural	First Nations living in Canada	All	Chronic Diseases and Comorbidities	18,542
75	Carlin	2022	Implementation of the 'Kimberley Mum's Mood Scale' across primary health care services in the Kimberley region of Western Australia: A mixed methods assessment	Australia	Rural	Aboriginal Australian	Female	Maternal and Child Health; Mental Health and Suicide Risk	548
76	Carlin	2022	Exploring Mental Health Presentations in Remote Aboriginal Community Controlled Health Services in the Kimberley Region of Western Australia Using an Audit and File Reviews	Australia	Rural	Aboriginal Australian	All	Mental Health and Suicide Risk	92
77	Campbell	2013	Pre-pregnancy predictors of hypertension in pregnancy among Aboriginal and Torres Strait Islander women in north Queensland, Australia; a prospective cohort study	Australia	Rural	Aboriginal Australian; Torres Strait Islander	Female	Maternal and Child Health	961
78	Campbell	2019	Childhood infection, antibiotic exposure and subsequent metabolic risk in adolescent and young adult Aboriginal Australians: practical implications	Australia	Rural	Aboriginal Australian	All	Other Health Conditions	343
79	Campbell	2022	Health care cost of crusted scabies in Aboriginal communities in the Northern Territory, Australia	Australia	Urban and Rural	Aboriginal Australian; Torres Strait Islander	All	Public Health and Prevention	42
80	Bruden	2015	Eighteen Years of Respiratory Syncytial Virus Surveillance: Changes in Seasonality and Hospitalization Rates in Southwestern Alaska Native Children	United States	Rural	Alaska Native	All	Infectious Diseases	5,966
81	Bradley	2020	Establishment of a sentinel surveillance network for sexually transmissible infections and blood borne viruses in Aboriginal primary care services across Australia: the ATLAS project	Australia	Urban and Rural	Aboriginal Australian; Torres Strait Islander	All	Public Health and Prevention	29
82	Bhat	2020	Psychosocial disadvantage and residential remoteness is associated with Aboriginal women's mental health prior to childbirth	Australia	Urban and Rural	Aboriginal Australian; Torres Strait Islander	Female	Maternal and Child Health	59,010
83	Best	2017	Genetic variants and risk of asthma in an American Indian population	United States	Rural	American Indian	All	Other Health Conditions	323
84	Bar-Zeev	2013	Use of Maternal Health Services by Remote Dwelling Aboriginal Women in Northern Australia and Their Disease Burden	Australia	Rural	Aboriginal Australian; Torres Strait Islander	Female	Healthcare Utilisation and Access	412
85	Askew	2019	Knowing our patients: a cross-sectional study of adult patients attending an urban Aboriginal and Torres Strait Islander primary healthcare service	Australia	Urban	Aboriginal Australian; Torres Strait Islander	All	Healthcare Utilisation and Access	400
